# Transcriptional regulation of miR-30a by YAP impacts PTPN13 and KLF9 levels and Schwann cell proliferation

**DOI:** 10.1016/j.jbc.2021.100962

**Published:** 2021-07-12

**Authors:** Alyssa Shepard, Sany Hoxha, Scott Troutman, David Harbaugh, Michael S. Kareta, Joseph L. Kissil

**Affiliations:** 1 Department of Molecular Medicine, The Scripps Research Institute, Jupiter, Florida, USA; 2 Department of Molecular Oncology, The Moffitt Cancer Center, Tampa, Florida, USA; 3 Genetics and Genomics Group, Sanford Research, Sioux Falls, South Dakota, USA

**Keywords:** Hippo pathway, yes-associated protein (YAP), miRNA, transcription coactivator, Schwann cells, AKT, protein kinase B, ChIP-seq, chromatin immunoprecipitation sequencing, hSC2λ, human Schwann, IRS-1, insulin receptor substrate, KD, knock-down, *KLF9*, Kruppel like factor 9, PI3K, phosphoinositide 3-kinases, *PTPN13*, protein tyrosine phosphatase non-receptor type 13, qPCR, quantitative PCR, TEAD, TEA domain, TFs, transcription factors, YAP, yes-associated protein

## Abstract

The Hippo pathway is a key regulatory pathway that is tightly regulated by mechanical cues such as tension, pressure, and contact with the extracellular matrix and other cells. At the distal end of the pathway is the yes-associated protein (YAP), a well-characterized transcriptional regulator. Through binding to transcription factors such as the TEA Domain TFs (TEADs) YAP regulates expression of several genes involved in cell fate, proliferation and death decisions. While the function of YAP as direct transcriptional regulator has been extensively characterized, only a small number of studies examined YAP function as a regulator of gene expression *via* microRNAs. We utilized bioinformatic approaches, including chromatin immunoprecipitation sequencing and RNA-Seq, to identify potential new targets of YAP regulation and identified miR-30a as a YAP target gene in Schwann cells. We find that YAP binds to the promoter and regulates the expression of miR-30a. Moreover, we identify several YAP-regulated genes that are putative miR-30a targets and focus on two of these, protein tyrosine pohosphatase non-receptor type 13 (*PTPN13)* and Kruppel like factor 9. We find that YAP regulation of Schwann cell proliferation and death is mediated, to a significant extent, through miR-30a regulation of *PTPN13* in Schwann cells. These findings identify a new regulatory function by YAP, mediated by miR-30a, to downregulate expression of *PTPN13* and Kruppel like factor 9. These studies expand our understanding of YAP function as a regulator of miRNAs and illustrate the complexity of YAP transcriptional functions.

The Hippo–Yes-associated protein (YAP) pathway has emerged as a central regulator of various processes, including cell proliferation, death, and fate (1, 2). The Hippo pathway is regulated by a host of stimuli including cell-surface receptors, G protein-coupled receptors, metabolic state, mechanical cues, and contact with the extracellular matrix and other cells (3, 4, 5). The core of the pathway is composed of a relatively well-defined kinase cascade, central to which are the Mst1/2 kinases that form a complex with the scaffold protein WW45 and phosphorylate Lats1/2 kinases. Phospho-Lats1/2, in complex with Mob1, bind to and phosphorylate YAP, a transcriptional coactivator, inhibiting YAP function by creating a binding site for 14-3-3 proteins and ultimately leading to YAP degradation (6). When hypophosphorylated, YAP enters the nucleus to form complexes with several transcriptional regulators to modulate the expression of extensive gene networks (5, 6).

Previous work has shown YAP can function both as a transcriptional repressor and activator (4, 5, 7, 8). As a transcriptional repressor, YAP has been shown to repress expression of mesendoderm lineage-specific genes in human embryonic stem cells (9). In addition, YAP can facilitate the recruitment of the NuRD complex, leading to histone deacetylation and repression of the expression of select target genes (4). We have previously shown that YAP can transcriptionally repress genes coding for the cyclin-dependent kinase inhibitor p27, a key mediator of cell contact inhibition (8). As a transcriptional activator, YAP has been shown to bind an extensive number of transcription factors (TFs), most notably the TEA domains (TEAD1-4). Work from several groups suggests the majority of YAP pro-proliferative and antiapoptotic functions are mediated through binding to the TEADs (10, 11, 12). The identity of specific YAP target genes is cell type and context dependent. Illustrative examples include breast cancer cells, where YAP/TEAD is found to be enriched along with the AP-1 complex at gene regulatory sites, driving oncogenic growth through activation of a wide network of target genes (13). In another example, in genetically engineered mouse models of KRAS-driven pancreatic ductal adenocarcinomas, the reemergence of tumors after withdrawal of oncogenic KRAS is driven by amplification of YAP. In these tumors, YAP/TEAD2 are shown to cooperate with E2F to activate transcriptional programs driving the cell cycle and DNA replication (14). As a final example, the assessment of Hippo–YAP function in *NF2*-deficient schwannoma identified that YAP regulation of gene expression drives cell survival and proliferation through an epidermal growth factor receptor–phosphoinositide 3-kinases (PI3K)–protein kinase B (AKT) signaling axis (7).

These studies have mostly focused on YAP targets genes that code for proteins with relatively well-characterized functions. However, it is unlikely that these targets alone represent the full complement of YAP’s activities. Indeed, multiple studies have demonstrated that YAP is localized to thousands of genomic locations from which it can regulate an extensive network of transcriptional activities (3, 4, 7, 8). To identify additional functions of YAP in Schwann cells, we analyzed YAP chromatin immunoprecipitation sequencing (ChIP-seq) data generated from actively proliferating Schwann cells and identified YAP enrichment in proximity to the promoters of several miRNA-coding genes (8). In contrast to the large body of work focusing on the direct regulation of protein-coding genes by YAP, relatively little is known about the direct regulation of miRNA expression or the regulation of miRNA processing by the Hippo–Yap pathway (15, 16, 17). Moreover, the limited number of studies published to date has demonstrated YAP may carry out opposing roles in miRNA processing. In our study, we focused on the direct regulation of miR-30a expression by YAP, which has been shown to be upregulated in schwannomas (18, 19). Our efforts demonstrate YAP is a direct transcriptional activator of miR-30a and identify two common targets of YAP and miR-30a, protein tyrosine phosphatase non-receptor type 1 (*PTPN13)* and Kruppel like factor 9 (*KLF9)*. Moreover, we find that the effects of YAP on Schwann cell proliferation are partially mediated through miR-30a regulation of *PTPN13*.

## Results

### YAP binds at the miR-30a promoter and regulates miR-30a expression

**Figure 1:**
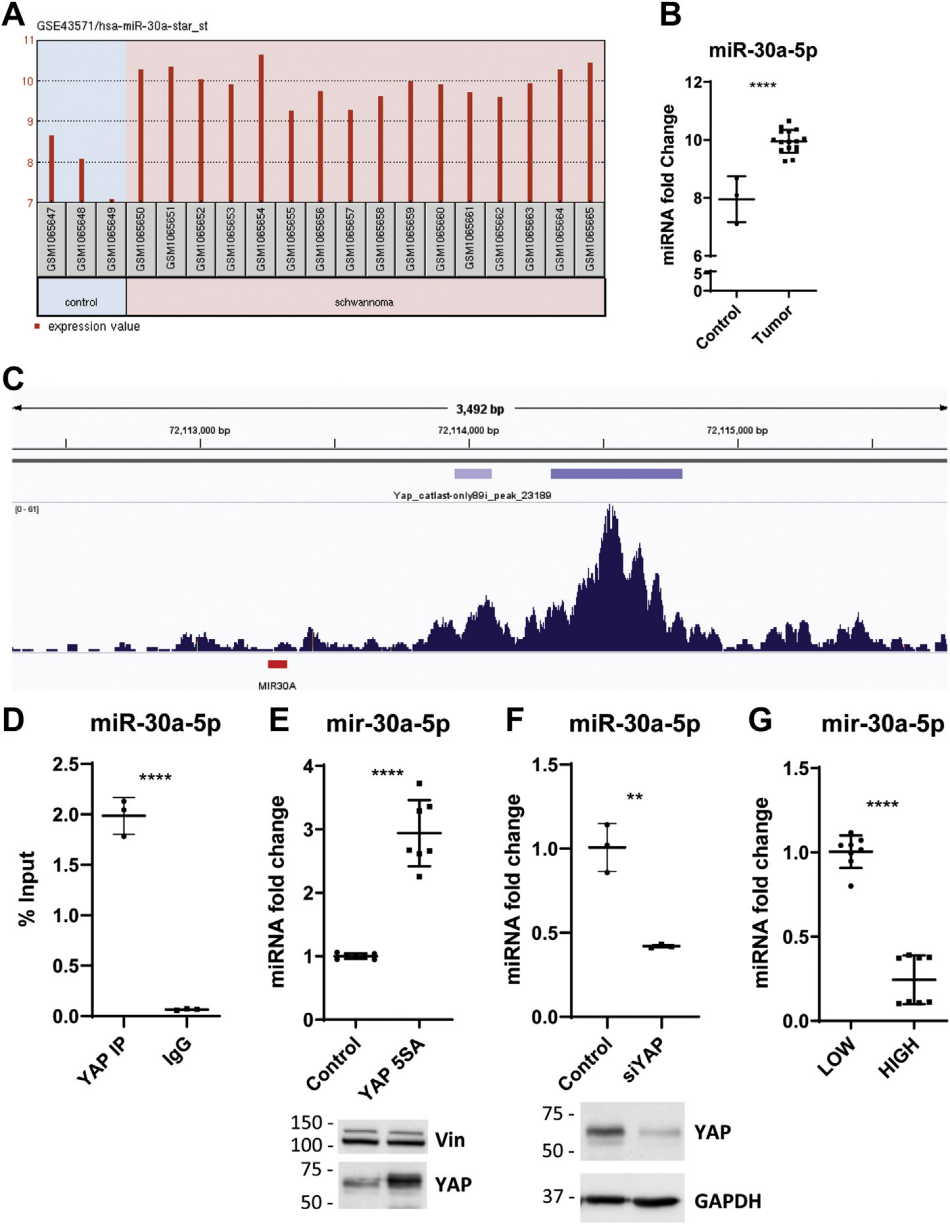
**Association and regulation of YAP and miR-30a.***A*, expression data for miR-30a and *B*, qPCR analysis of miR-30a mRNA levels from schwannoma samples from GSE43571 (18). Each *dot* represents a separate tumor sample (∗∗∗∗*p* < 0.0001, two-tailed Student’s *t* test; error bars = SD). *C*, gene browser tracks of YAP ChIP at the *miR-30a* promoter. YAP peaks are indicated by *purple bars* above the peak representations. miR-30a genomic location is indicated in *red*. *D*, assessment of miR-30a enrichment by YAP ChIP-qPCR. IgG used as a negative control. Representative image of three individual experiments with three replicates each (n = 3; ∗∗∗∗*p* < 0.0001, two-tailed Student's *t* test; error bars = SD). *E*, analysis of miR-30a expression levels in hSC2λ cells transfected with a control vector or YAP-5SA expression vector. Representative image of three individual experiments with seven replicates each (n = 3; ∗∗∗∗*p* < 0.0001, two-tailed Student's *t* test; error bars = SD). Western blot confirms overexpression of YAP. Vinculin (vin) was used as a loading control. Molecular weight markers are indicated in kilodalton. *F*, analysis of miR-30a expression levels in hSC2λ cells transfected with siRNA nontargeting control or siRNA-targeting YAP. Representative image of three individual experiments with three replicates each (n = 3; ∗∗*p* < 0.01, two-tailed Student's *t* test; error bars = SD). Western blot confirms downregulation of YAP. GAPDH was used as a loading control. Molecular weight markers are indicated in kilodalton. *G*, effect of cell density on miR-30a assessed by qPCR. Representative image of three individual experiments with six replicates each (n = 3; ∗∗∗∗*p* < 0.0001, two-tailed Student's *t* test; error bars = SD). ChIP-seq, chromatin immunoprecipitation sequencing; hSC2λ, human Schwann; IgG, immunoglobulin G; qPCR, quantitative PCR; YAP, yes-associated protein.

We previously used ChIP followed by next-generation sequencing to identify genomic regions to which YAP binds in actively proliferating human Schwann (hSC2λ) cells (8). During this effort, we identified a total of 7019 peaks, including peaks at the promoters of previously identified YAP target genes such as *CTGF*, *TEAD1*, *AMOTL2*, and *ANKRD1*. Along with the known target genes, we identified a number of peaks in close proximity to miRNA-coding genes that have not been previously identified as YAP target genes (Table S3). Of the identified genes, we focused on miR-30a as it is found to be overexpressed in multiple human schwannoma tumor samples (Fig. 1, *A* and *B*) (18). We directly assessed YAP binding to the *miR-30a* promoter by ChIP–quantitative PCR (qPCR) and observed a significant enrichment of YAP at the *miR-30a* promoter compared with an immunoglobulin G control (Fig. 1, *C* and *D*). This enrichment suggests that regulation of miR-30a expression could be mediated by YAP, at the transcriptional level. We tested this hypothesis by overexpression of YAP in hSC2λ cells by transfection of the cells with an expression vector for YAP-5SA, a constitutively active form of YAP. The YAP-5SA-overexpressing cells showed a significant increase in miR-30a expression compared with cells transfected with a control vector (Fig. 1*E*). Overexpression of endogenous YAP (YAP WT) also led to a significant increase in miR-30a expression (Fig. S1*A*). To assess the consequences of YAP loss, we used siRNA to knock-down (KD) YAP levels. This led to a significant decrease in miR-30a expression (Fig. 1*F*). Furthermore, as YAP activity is mediated by cell density, we assessed the impact of cell density on miR-30a expression (5). At high cell density, where YAP is excluded from the nucleus, miR-30a levels were also decreased when compared with cells at low density where YAP is nuclear and active (Fig. 1*G*). This decrease in miR-30a can be rescued through overexpression of YAP-5SA at high cell density (Fig. S1*B*). These data support the hypothesis that YAP regulates miR-30a expression at the transcriptional level by binding at the miR-30a promoter region.

### Identification of YAP-regulated miR-30a-binding targets

**Figure 2:**
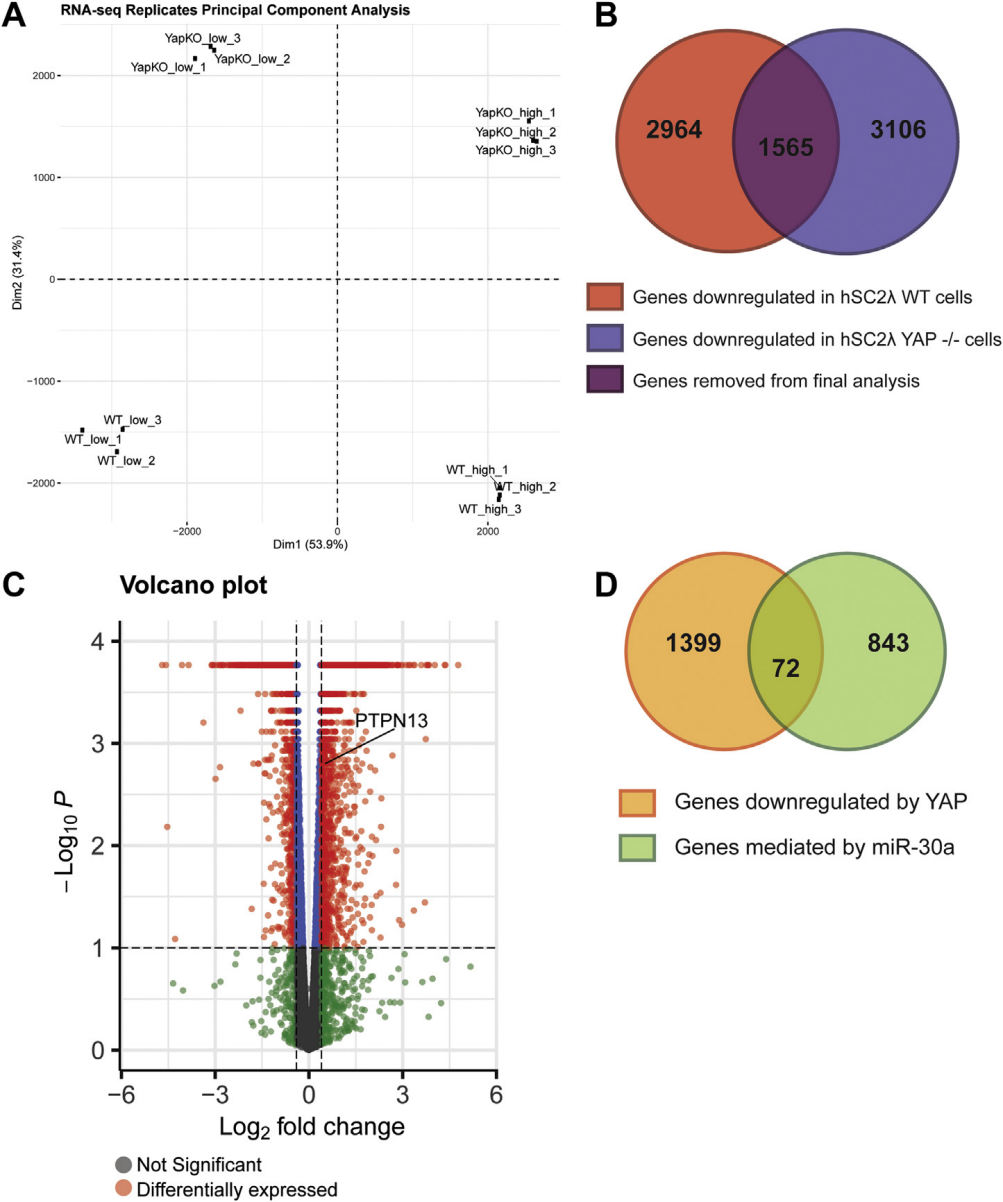
**Identifying genes mediated by both YAP and miR-30a.***A*, PCA plot of RNA-Seq replicates. The replicates include hSC2λ WT grown at high and low densities and hSC2λ YAP −/− (YapKO) grown at high and low densities. *B*, graphic representation of how genes downregulated by YAP were identified. RNA-Seq analysis identified 2964 downregulated genes in hSC2λ WT cells (*red*) and 3106 downregulated genes in hSC2λ YAP −/− cells (*blue*). 1565 genes were removed from the WT downregulated genes (*purple*) to identify 1399 genes that are likely downregulated specifically by YAP. *C*, volcano plot showing differentially expressed genes in hSC2λ cells at high and low densities as determined by RNA-Seq analysis. *Red* indicates genes that are downregulated in a YAP-dependent manner. PTPN13 is highlighted on the *graph*. *D*, schematic showing the overlap between genes downregulated by YAP and genes mediated by miR-30a. hSC2λ, human Schwann; PCA, principal component analysis; YAP, yes-associated protein.

To identify potential miR-30a targets, we interrogated the miR database (mirdb.org) and TargetScan (targetscan.org), which provided us with 843 predicted binding targets (Table S4). To narrow the list of candidate targets, we used RNA-Seq analysis to identify genes that are transcriptionally downregulated by YAP. Next-generation sequencing was performed on RNA extracted from hSC2λ WT and hSC2λ YAP −/− cells plated at high and low cell densities. Log2 fold changes were calculated between the hSC2λ WT high and low densities and hSC2λ YAP −/− high and low densities. Genes that overlapped between the two analyses were removed from the final list of downregulated genes, as they are likely not regulated primarily by YAP if their expression changed in YAP −/− cells. This analysis provided us with 1399 genes that are selectively downregulated when YAP is present and transcriptionally active (Table S5 and Fig. 2, *A*–*C*). Using these two lists, we identified 72 targets that are overlapping with candidate miR3-30a targets (Table S6 and Fig. 2*D*). Gene ontology functional annotation analysis of these 72 genes did not result in a clear classification. Furthermore, we examined these genes to determine whether there were any TEAD-binding motifs in close proximity. This motif analysis did not identify any TEAD motifs, suggesting that the identified genes do not include direct targets of YAP, but rather YAP targets that are regulated through indirect means such as miRNAs. From this list, we chose to focus on PTPN13 because it has been previously shown to have tumor-suppressive functions (20, 21, 22). In addition, to increase the robustness of our analysis, we selected KLF9 as a second potential target as it was identified as downregulated by YAP in an independent study conducted in MCF-10A cells and previously suggested to have tumor-suppressive activities (4, 23, 24, 25).

### YAP and miR-30a levels regulate transcriptional expression of two targets, PTPN13 and KLF9

**Figure 3:**
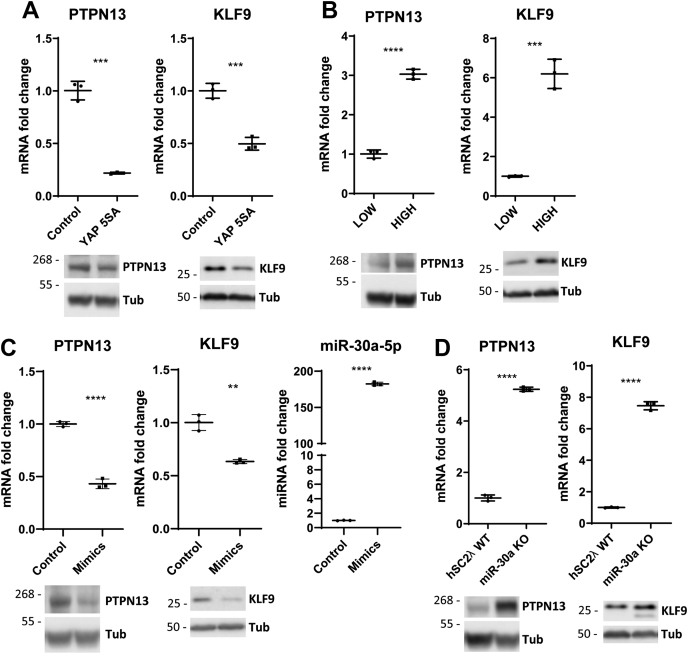
**Transcriptional regulation of PTPN13 and KLF9 expression by YAP and miR-30a.***A*, qPCR analysis of PTPN13 and KLF9 expression upon overexpression of YAP-5SA or control vector. Western blots show correlation of mRNA and protein expression. Tubulin (Tub) was used as a loading control. Molecular weight markers are indicated in kilodalton. See Figure 1*E* for corresponding YAP Western blots (n = 3; ∗∗∗*p* < 0.001, two-tailed Student’s *t* test; error bars = SD). *B*, qPCR analysis of PTPN13 and KLF9 expression at high and low cell densities. Western blots show correlation of RNA and protein expression. Tubulin (Tub) was used as a loading control. Molecular weight markers are indicated in kilodalton. See Fig. S1*B* for corresponding YAP Western blots (n = 3; ∗∗∗∗*p* < 0.0001, ∗∗∗*p* < 0.001, two-tailed Student's *t* test; error bars = SD). *C*, qPCR analysis of PTPN13 and KLF9 with miR-30a overexpression using miRNA mimics. miRIDIAN miRNA mimic negative controls were used as a transfection control. Western blots show correlation of RNA and protein expression. Tubulin (Tub) was used as a loading control. Molecular weight markers are indicated in kilodalton. miRNA qPCR confirms upregulation of miR-30a expression (n = 3; ∗∗*p* < 0.01, ∗∗∗∗*p* < 0.0001, two-tailed Student's *t* test; error bars = SD). *D*, qPCR analysis of PTPN13 and KLF9 in hSC2λ WT and miR-30a CRISPR KO cells. Western blots show correlation of RNA and protein expression. Tubulin (Tub) was used as a loading control. Molecular weight markers are indicated in kilodalton (n = 3; ∗∗∗∗*p* < 0.0001, two-tailed Student's *t* test; error bars = SD). *A*–*D*, all qPCRs are representative of three independent experiments with three replicates each. hSC2λ, human Schwann; qPCR, quantitative PCR; YAP, yes-associated protein.

To confirm the relationship between YAP, miR-30a, PTPN13, and KLF9, we assessed target expression levels using qPCR and Western blotting. First, hSC2λ cells were transfected with YAP-5SA or a control vector, and the expressions of PTPN13 and KLF9 were compared. Expression levels of both these genes were significantly decreased upon YAP-5SA overexpression (Fig. 3*A*). These effects were also seen upon overexpression of WT YAP (Fig. S2*A*). Furthermore, at high cell density when YAP activity is decreased, PTPN13 and KLF9 expression increased significantly (Fig. 3*B*). Attenuation of YAP using siRNAs led to a significant increase in PTPN13 and KLF9 expression (Fig. S2*B*). In a second approach, the overexpression of miR-30a mimics led to a decrease in PTPN13 and KLF9 levels (Fig. 3*C*). To assess the consequences of miR-30a loss of function, we used CRISPR-mediated genome editing to generate miR-30a KO hSC2λ cells (Fig. S3, *A* and *B*). Inactivation of miR-30a led to a significant increase in both PTPN13 and KLF9 levels (Fig. 3*D*). Western blots of all experiments confirmed the trends seen in qPCRs (Fig. 3). These data indicate that both YAP and miR-30a regulate the levels of PTPN13 and KLF9.

### Loss of miR-30a or mutation of miR-30a-binding sites negates YAP transcriptional regulation of PTPN13 and KLF9

**Figure 4:**
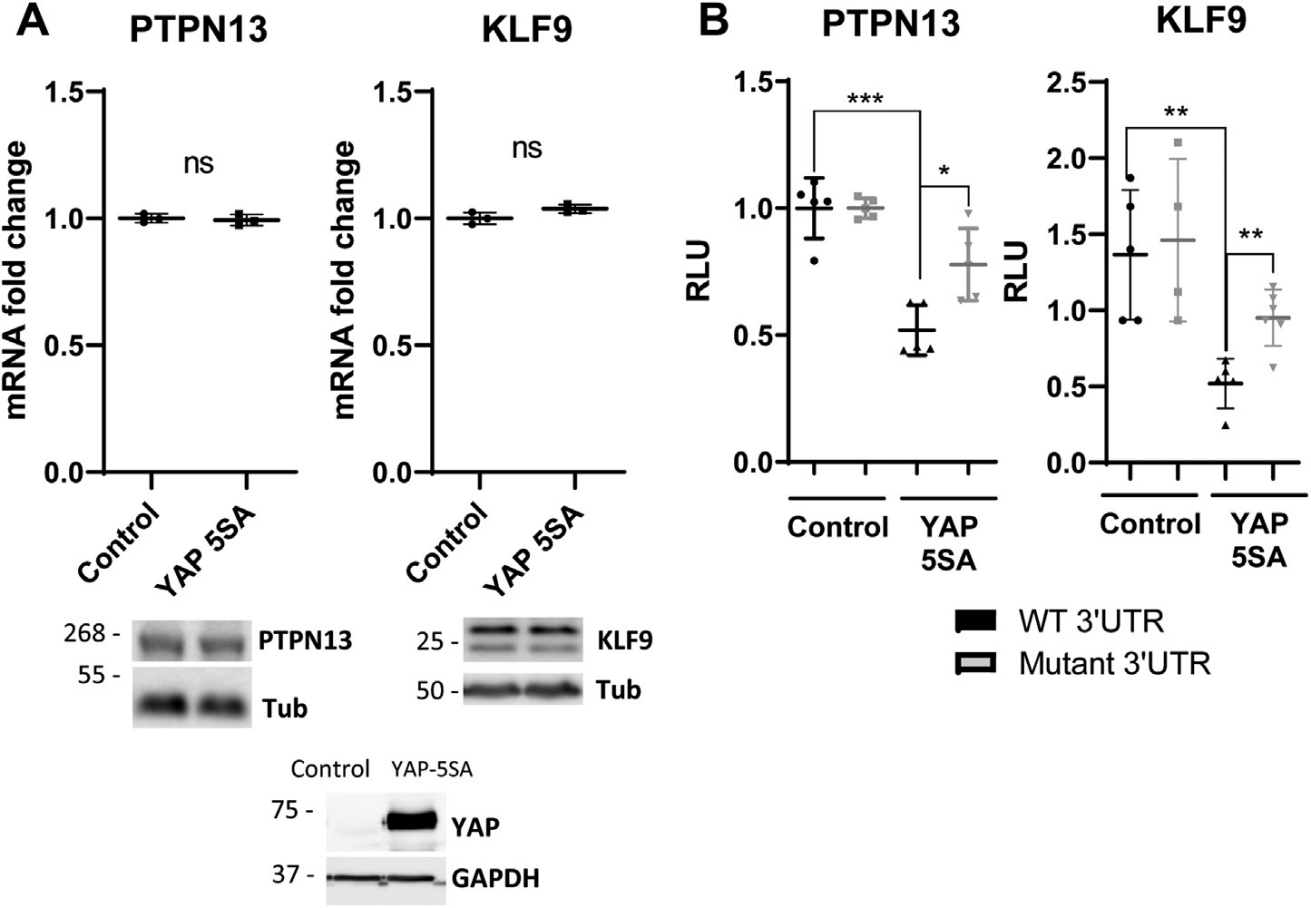
**Dual-Glo luciferase assay for association of miR-30a with 3′UTR binding sites.***A*, qPCR analysis of PTPN13 and KLF9 expression in miR-30a KO cells (hSC2λ) upon YAP-5SA overexpression. Control indicates an empty control vector. Representative image of three individual experiments with three replicates each (n = 3; two-tailed Student's *t* test; error bars = SD). Western blots corroborate qPCR analyses and confirms YAP upregulation (the panel showing YAP-5SA expression is the same as in Fig. 5*E* because the analysis was done using the same cells). Tubulin (Tub) and GAPDH were used as loading controls. Molecular weight markers are indicated in kilodalton. *B*, YAP −/− cells (hSC2λ) were cotransfected with a pmiR-GLO plasmid with a control vector or YAP-5SA. pmiR-GLO contained the WT 3′UTR (*black*), or 3′UTR with mutated miR-30a-binding sites (*gray*) of PTPN13 and KLF9. Luminescence was measured for each condition to assess binding of miR-30a. Representative image of three individual experiments with five replicates each (n = 3; ∗*p* < 0.05, ∗∗*p* < 0.01, ∗∗∗*p* < 0.001, two-tailed Student's *t* test; error bars = SD). hSC2λ, human Schwann; ns, not significant; qPCR, quantitative PCR; RLU, relative light units; YAP, yes-associated protein.

To determine whether the YAP regulation of PTPN13 and KLF9 is mediated through miR-30a, we examined the consequences of YAP-5SA overexpression on PTPN13 and KLF9 levels in miR-30a KO cells. Although YAP-5SA overexpression in hSC2λ cells WT for miR-30a results in significant reduction in the levels of PTPN13 and KLF9 levels (Fig. 3*A*), overexpression in the miR-30a-deficient cells had no significant effect on levels of either PTPN13 or KLF9 as shown *via* qPCR and Western blot (Fig. 4*A*). In addition, we used a luciferase reporter assay in which the 3′UTRs of the PTPN13 and KLF9 mRNAs, which harbor the miR-30a-binding sites, were cloned in an expression vector downstream of a luciferase reporter gene, *luc2* (Fig. S4). Cotransfection of YAP-5SA along with the expression plasmid containing the WT 3′UTRs and luciferase reporter resulted in a significant decrease in luciferase activity levels for both PTPN13 and KLF9 (Fig. 4*B*). In contrast, the cotransfection of YAP-5SA with the luciferase reporter plasmids that carry 3′UTRs where the miR-30a-binding site has been mutated resulted in a significant rescue of luciferase activity levels (Fig. 4*B*). These experiments suggest that YAP regulation of PTPN13 and KLF9 is mediated to a significant extent through miR-30a, as YAP cannot suppress luciferase activity to the same extent when the miR-30a-binding sites in the 3′UTRs were mutated.

### Loss of miR-30a results in slower growth phenotype and is rescued by reintroducing miR-30a expression or KD of PTPN13

**Figure 5:**
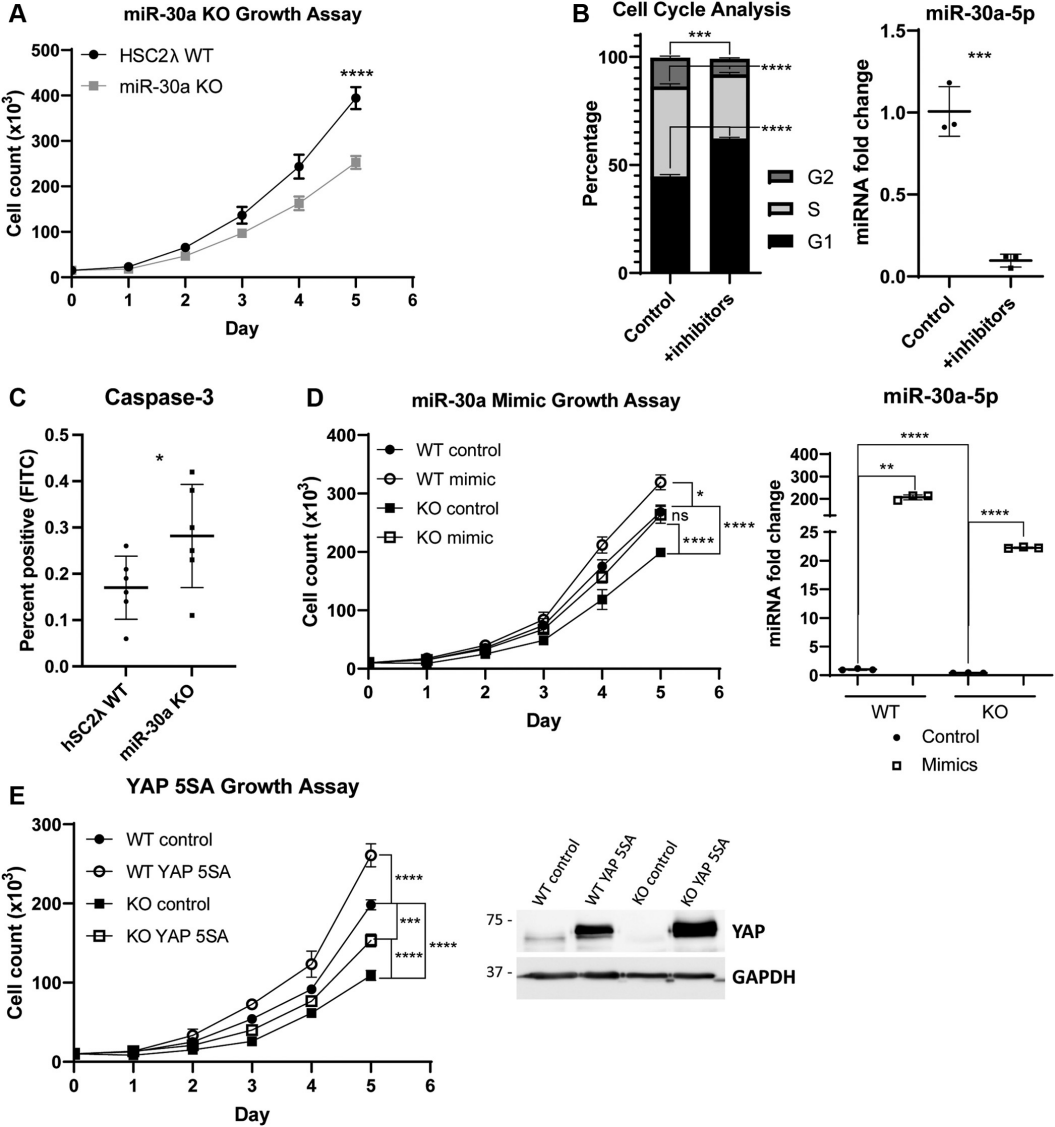
**Growth rate differences between hSC2λ WT and miR-30a KO cells and subsequent rescue of phenotype.***A*, growth rates of hSC2λ WT (*black circles*) and miR-30a KO (*gray squares*) cells were assessed over 5 days by cell counting. Significance was calculated for day 5 values (n = 3; ∗∗∗∗*p* < 0.0001, two-tailed Student’s *t* test; error bars = SD). *B*, hSC2λ WT cells were transfected with miR-30a inhibitors or miRNA inhibitor negative controls. Cells were stained with propidium iodide and analyzed *via* flow cytometry. The cell cycle was determined by peak analysis of the propidium iodide histograms. qPCR analysis confirms knock-down of miR-30a. Replicates are indicated by individual data points (n = 3; ∗∗∗*p* < 0.001, ∗∗∗∗*p* < 0.0001, two-tailed Student’s *t* test; error bars = SD). *C*, hSC2λ WT and miR-30a KO cells were stained with anti-Caspase-3 conjugated to FITC. The percentage of FITC-positive cells was determined with flow cytometry. Replicates are indicated by individual data points (n = 2; ∗*p* < 0.05, two-tailed Student’s *t* test; error bars = SD). *D*, WT (*circle*) and KO (*square*) cell lines (hSC2λ) were transfected with miRNA mimic negative control (control; *black symbol*) or miR-30a mimics (mimic; *white symbol*). Growth rates were assessed over 5 days by cell counting. Significance was calculated using three-way ANOVA on transformed data. The differences in growth rates due to the interaction of the genotype and treatment was significant (∗*p*-value = 0.0296). Post hoc analysis was used to determine significance of day 5 values (n = 3; ∗*p* < 0.05, ∗∗∗∗*p* < 0.0001; error bars = SD). qPCR analysis confirms upregulation of miR-30a using mimics (n = 3; ∗∗*p* < 0.01, ∗∗∗∗*p* < 0.0001, two-tailed Student’s *t* test; error bars = SD). *E*, WT (*circle*) and KO (*square*) cell lines (hSC2λ) were transfected with a control vector (control; *black symbol*) or YAP-5SA (*white symbol*). Growth rates were assessed over 5 days by cell counting. Significance was calculated using three-way ANOVA on transformed data. The difference in growth rates due to the interaction of the genotype and treatment was not significant (*p*-value = 0.9425), but the individual effect of the genotype (∗∗∗∗*p*-value < 0.0001) and treatment (∗∗∗∗*p*-value < 0.0001) was significant. Post hoc analysis was used to determine significance of day 5 values (n = 3; ∗∗∗*p* < 0.001, ∗∗∗∗*p* < 0.0001; error bars = SD). Western blot confirms upregulation of YAP. GAPDH was used as a loading control. Molecular weight markers are indicated in kilodalton. *A*, *B*, *D*–*E*, growth assays and qPCRs shown are representative of three independent experiments with three replicates each. hSC2λ, human Schwann; ns, not significant; qPCR, quantitative PCR; YAP, yes-associated protein.

After establishing the relationship between YAP, miR-30a, PTPN13, and KLF9, we next sought to determine the consequences of miR-30a loss at the cellular level. To this end, we performed cell-counting experiments in the miR-30a KO hSC2λ cells. The inactivation of miR-30a resulted in reduced cell numbers over time (Fig. 5*A*). This same trend is seen upon miR-30a KD in hSC2λ cells transfected with miR-30a inhibitors (Fig. S5*A*). In addition, the reduction in the growth rate is corroborated by cell cycle analysis of hSC2λ cells transfected with miR-30a inhibitors, in which cells treated with inhibitors show significantly more cells in G1 and fewer cells in the G2 and S phases than the control (Fig. 5*B*). In addition, we observed an increase in activated caspase-3 in the miR-30a KO cells compared with hSC2λ WT, suggesting an increase in cells undergoing apoptosis upon loss of miR-30a (Fig. 5*C*). The growth rate is restored if miR-30a is reintroduced into the KO cells using miR-30a mimics (Fig. 5*D*). Interestingly, this result also indicated there is a slight increase in growth rates in the WT cells that have miR-30a overexpressed. We observed this same phenomenon in HEI-193 cells, in which growth is slowed upon KD of miR-30a and is rescued upon reintroduction of miR-30a (Fig. S5*B*). In agreement with previous studies, the overexpression of YAP-5SA increases growth rates in WT cells (8). However, the overexpression of YAP-5SA in the miR-30a KO cells showed only a partial recovery in growth rates when compared with miR-30a WT cells (Fig. 5*E*). These results were also seen in miR-30a KD cells (Fig. S5*C*).

**Figure 6:**
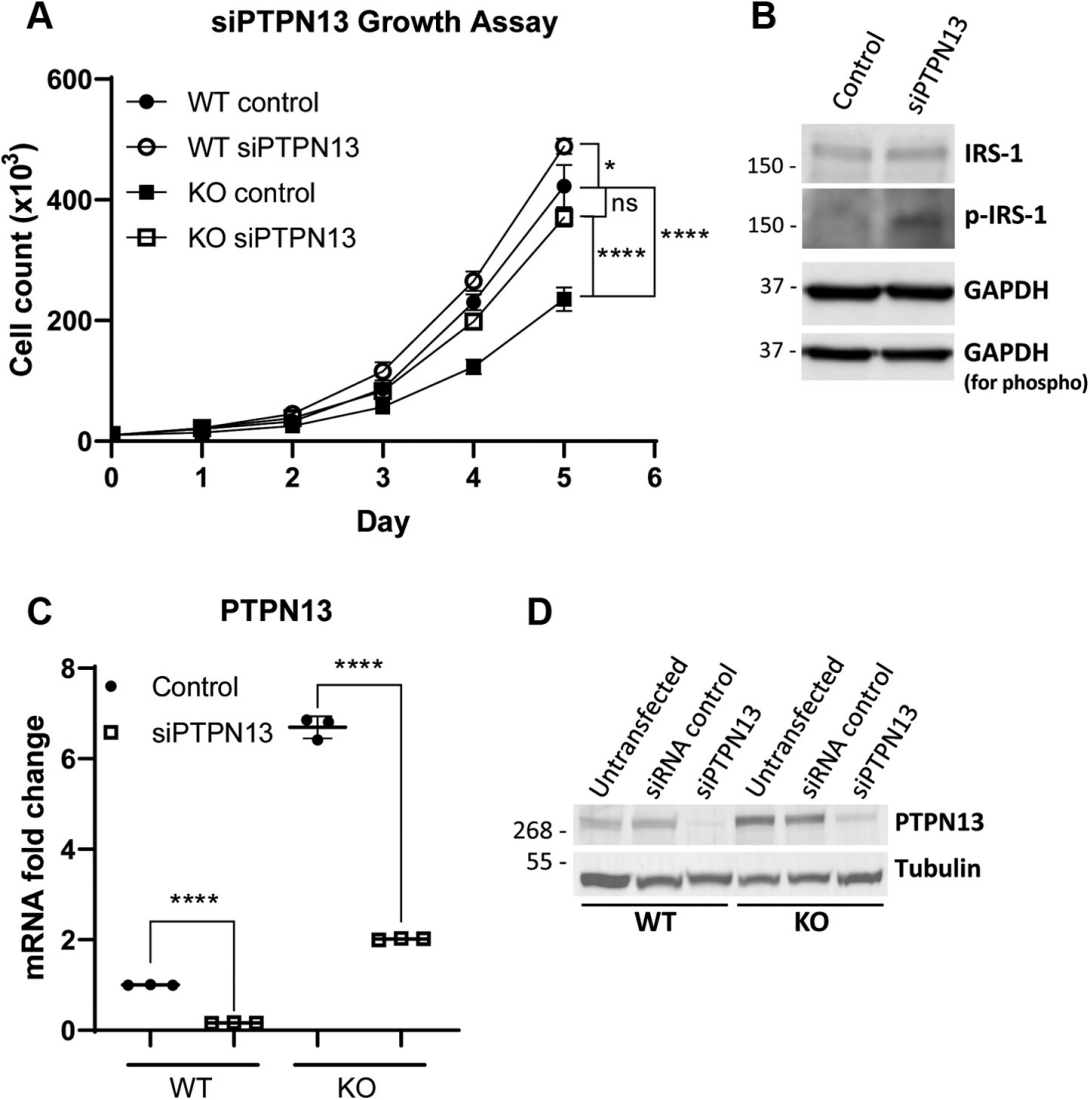
**Effect of PTPN13 on growth rates in miR-30a KO cells.***A*, WT (*circle*) and KO (*square*) cell lines (hSC2λ) were transfected with a nontargeting control siRNA (control; *black symbol*) or siRNA-targeting PTPN13 (*white symbol*). Growth rates were assessed over 5 days by cell counting. Significance was calculated using three-way ANOVA on transformed data. The difference in growth rates due to the interaction of the genotype and treatment was significant (∗∗∗∗*p*-value < 0.0001). Post hoc analysis was used to determine significance of day 5 values (n = 3; ∗*p* < 0.05, ∗∗∗∗*p* < 0.0001; error bars = SD). Representative of three independent experiments with three replicates each. *B*, hSC2λ cells were transfected with nontargeting control siRNA or siPTPN13. Protein was extracted and used to assess total IRS-1 and phospho-IRS-1 (p-IRS-1, Tyr612) expression. GAPDH was used as a loading control. Molecular weight markers are indicated in kilodalton. *C*, qPCR analysis for PTPN13 expression to show knock-down efficiency of siPTPN13 on PTPN13 mRNA in hSC2λ WT or miR-30a KO cells. Representative of three independent experiments with three replicates each (n = 3; ∗∗∗∗*p* < 0.0001, two-tailed Student’s *t* test; error bars = SD). *D*, Western blot analysis to show efficiency of PTPN13 knock-down in hSC2λ WT or miR-30a KO cells. Untransfected cells are used as an additional control. siRNA control indicates the nontargeting control. Tubulin was used as a loading control. Molecular weight markers are indicated in kilodalton. hSC2λ, human Schwann; ns, not significant; qPCR, quantitative PCR.

Finally, we used siRNA to knock down the expression of PTPN13 or KLF9. Our results show that decreasing PTPN13 expression through siRNA KD rescues the slower growth phenotype seen in the miR-30a KO cells (Fig. 6*A*). KD of PTPN13 also results in a slight growth rate increase in hSC2λ WT cells. We observed the same trends in growth rates upon testing individual siRNAs against PTPN13 (Fig. S6). In contrast, KD of KLF9 did not affect growth rates in either the miR-30a KO or WT cells (Fig. S7). In addition, we investigated the influence of PTPN13 on the PI3K/Akt pathway. Previous studies have established a connection between PTPN13 and the phosphorylation status of insulin receptor substrate 1 (IRS-1) (26, 27). These studies also confirm the role of PTPN13 as a tumor suppressor in regulating apoptosis and tumor aggressiveness through IRS-1 (26, 27). Upon KD of PTPN13 using siRNAs, we observed an increase in phosphorylation of IRS-1 at Tyr612 (Fig. 6*B*). This suggests the growth-suppressive role of PTPN13 we observed may be functioning through IRS-1 signaling and requires further investigation. Taken together, these data demonstrate that loss of miR-30a results in a slower growth phenotype that can be rescued by reintroduction of miR-30a, leading to decreased PTPN13 expression. In addition, our results suggest KLF9 does not play a significant role in Schwann cell growth, at least in relation to miR-30a. We also show that overexpression of YAP-5SA can partially rescue the growth phenotype observed in the miR-30a KO cells. This result is not surprising, as YAP mediates cell growth through a variety of pathways outside of miR-30a (3, 7, 8, 28, 29).

## Discussion

While the role of YAP as direct transcriptional regulator has been extensively characterized, only a small number of studies have examined YAP function as a regulator gene expression *via* miRNAs (1, 2, 3, 4, 5, 6, 7, 8). Previous studies have led to contradicting conclusions regarding YAP’s function, with proposed mechanisms ranging from global regulation of miRNA processing to regulation of individual miRNA species (15, 16, 17). As an example, the nuclear accumulation of YAP/TAZ in MCF10A cells growing at low cell density led to an increase of several miRNAs. This was thought to be mediated through indirect YAP regulation of the miRNA processor, Dicer, *via* the Let-7 miRNA (15). In another study, nuclear YAP was shown to bind p72, a central regulator of miRNA processing (16). This binding prevents association of p72 with the microprocessor, thus impeding processing of pre-miRNAs and leading to global downregulation of miRNAs (16). The differences in proposed mechanisms could reflect the use of different cell types in these studies.

Although the aforementioned studies establish a relationship between YAP localization and miRNAs, the effects of YAP on other regulatory mechanisms involving direct transcriptional regulation of miRNAs are less understood. To date, only a handful of miRNAs directly regulated by YAP has been identified (30, 31, 32, 33, 34, 35, 36, 37). The bantam miRNA, an important regulator of tissue growth in *Drosophila*, was the first miRNA to be identified as a direct target of YAP’s (Yorkie, Yki, in *Drosophila*) transcriptional activity (30, 31, 32). In addition, Yorkie has been linked to regulation of the miR-2 family, which includes miR-2a and miR-2b (33). Outside of *Drosophila*, the miR-29 family has been identified as a target of YAP (34, 35). Interestingly, this YAP–miR-29 relationship was linked to the regulation of the tumor suppressor PTEN, suggesting a role for YAP–miRNA relationships in cancer development (34, 35). Additional miRNAs shown to be transcriptionally regulated by YAP include miR-130a, miR-206, miR-296, miR-25, miR-93, and miR-106 (36, 37, 38, 39). Our study highlights a previously unknown direct relationship between YAP and the transcriptional regulation of miR-30a, thus contributing to accumulating evidence for an additional transcriptional role of YAP outside the regulation of protein-coding genes.

We focused on the relationship between YAP and miR-30a in Schwann cells because of the fact that miR-30a was found to be upregulated in human schwannomas, suggesting miR-30a overexpression could play a role in the development of these tumors (18, 19). Our findings demonstrate binding of YAP to the promoter of miR-30a and confirmed the expression of miR-30a to be induced by YAP expression. Although we did not directly assess the TFs through which YAP might serve to regulate miR-30a, we conducted a bioinformatic analysis of the genomic region surrounding the YAP-enriched peaks in the vicinity of the miR30a gene using GeneHancer (40). This analysis identified a promoter/enhancer (GH06J071400) in close proximity to miR30a, positioned 3.5 kB from the transcriptional start site. This region included binding sites for several TFs, including consensus binding sites for the TEADs, which are major binding partners of YAP. This suggests that YAP regulation of miR30a is mediated *via* the TEADs; however, this would need to be confirmed experimentally.

Moreover, we observed a decrease in cell growth rates and an increase in cells undergoing apoptosis upon loss of miR-30a and subsequently observed increased proliferation in cells with miR-30a overexpression. These data suggest miR-30a has a role in regulating Schwann cell proliferation. Overexpression of YAP in the miR-30a–deficient cells did not fully recapitulate the consequences of YAP overexpression in miR-30a WT cells. This suggests that while YAP’s effects on cell growth are mediated to a significant extent *via* miR-30a, additional mechanisms that are miR-30a independent play a role. This observation is in line with what is already known about YAP regulation of Schwann cell growth (28, 29).

It should be noted that there are a number of studies showing miR-30a can act as a tumor suppressor. These studies show miR-30a expression to inhibit lung cancer cell growth, prevent gastric cancer in mice, and inhibit epithelial–mesenchymal transition in conjunction with p53 (41, 42, 43, 44). This evidence suggests miR-30a could have opposing roles in cancer, which are likely to be cell-type dependent. Using RNA-Seq, we identified multiple potential overlapping targets of miR-30a and YAP. For the purposes of this study, we focused on two genes, *PTPN13* and *KLF9*, and found that both YAP and miR-30a overexpression leads to a downregulation of these two targets and that these effects of YAP required miR-30a. In regard to cell proliferation, we observed that KD of PTPN13 in miR-30a-deficient cells rescued the lower cell growth rate phenotype and led to a significant increase in growth rates in WT cells. In addition, we observed an increase in phosphor-IRS-1 upon downregulation of PTPN13, in line with previous studies suggesting PTPN13 functions through IRS-1 (26, 27). These data suggest that PTPN13 may be a negative regulator of cell growth in Schwann cells and achieve this regulation through the PI3K/Akt pathway. This observation is in line with the previous studies suggesting tumor-suppressive roles of PTPN13 (20, 21, 22). PTPN13 is a member of the protein tyrosine phosphatase family and has previously been shown to have tumor-suppressive functions (20, 21, 22). Specifically, PTPN13 has been shown to be involved in a negative feedback mechanism with Her2 activity, affecting the invasiveness of Her2 tumor cells (20). PTPN13 overexpression was also correlated to lower cell proliferation rates in hepatocellular carcinoma and clear renal cell carcinoma (21, 22). Further studies are required to identify the mechanisms through which PTPN13 might regulate Schwann cell proliferation. KLF9 is a member of the Krüppel-like factor family, which are DNA-binding TFs that affect a wide range of biological processes (45). KLF9 and its role in cancer has not been extensively investigated, although some studies have suggested KLF9 plays a tumor-suppressive role (23, 24, 25). Indeed, KLF9 KO mice show increased incidence of colorectal cancer (23). KLF9 overexpression in pancreatic cells decreased proliferation rates, inhibited migration and invasion, and induced apoptosis (24). Suppression of growth and stabilization of p53 was observed in hepatocellular carcinoma cells upon restoration of KLF9 expression (25). However, the KD of KLF9 did not have a significant effect on growth rates in miR-30a KO or WT cells, suggesting KLF9 may not have a significant role in the regulation of Schwann cell growth.

## Experimental procedures

### Cell lines

The HEI-193 human schwannoma cells were obtained as described previously (7). hSC2λ cells were obtained from the laboratory of Dr Margaret Wallace (46). All cell lines were authenticated by short tandem repeat profiling (DDC Medical). Cells were maintained as previously described (8)

### Plasmids, siRNA, and miRNA mimics and inhibitors

The pCMV-Flag-YAP-5SA (#27371), PX459 (#62988), and miR-30a (#20670) plasmids were purchased from Addgene (www.addgene.org). The ON-TARGETplus Human PTPN13 (5783) SMARTpool siRNA (L-008065-00-0005), individual ON-TARGETplus Human PTPN13 siRNAs (J-008065-05-0002 and J-008065-06-0002), ON-TARGETplus Human KLF9 (687) SMARTpool siRNA (L-0112233-00-0005), ON-TARGETplus Human YAP1 SMARTpool siRNA (L-012200-00-0005), ON-TARGETplus nontargeting control pool (D-001810-10-05), miRIDIAN miRNA hsa-mir-30a-3p Mimic (C-300506-03-0005), miRIDIAN miRNA hsa-mir-30a-5p Mimic (C-300505-03-0005), miRIDIAN miRNA Mimic Negative Controls #1 and #2 (CN-001000-01-05 and CN-002000-01-05), miRIDIAN miRNA hairpin inhibitor for has-miR-30a-3p and has-miR-30a-5p (IH-300506-05-0002 and IH-300505-05-0002), and miRIDIAN miRNA hairpin inhibitor negative controls #1 and #2 (IN-001005-01-05 and IN-002005-01-05) were all purchased from GE Healthcare Dharmacon, Inc. Unless otherwise specified, 2.5 μg of plasmid was used for transfections. siRNAs and mimics were used at a final concentration of 20 nM for transfections.

### Antibodies

All antibodies used in Western blot analysis and ChIP are listed in Table S1.

### Transfections

Plasmid transfections were performed using Lipofectamine 2000 (Life Technologies, Inc). For siRNA, miRNA mimics, and miRNA inhibitors, Lipofectamine RNAiMax (Life Technologies, Inc) was used according to the manufacturer instructions. Media were changed 4 h after transfection. Cells were analyzed and used in subsequent experiments 24 h after transfection.

### ChIP and quantitative real-time PCR analysis

ChIP was performed as previously described (8). The YAP antibody used in the immunoprecipitation was from Cell Signaling Technology (#4912). DNA was used for real-time PCR using the SYBR Green PCR kit. A standard dilution curve was obtained for each input, and 1 μl of ChIP DNA was used in each PCR. Melt curves were analyzed to confirm specificity of the amplified target.

### ChIP-seq library preparation, sequencing, and analysis

The ChIP-seq libraries were prepared using the NEBNext Ultra II DNA Library Prep Kit for Illumina (NEB) and sequenced using the NextSeq 500 High Output v2 Kit (Illumina) on the NextSeq 500 platform as previously described (8). Data processing was carried out as previously described (8). Briefly, cutadapt v1.8.1 was used to remove adaptor sequences and low-quality end reads (47). Reads were aligned to the hg19 genome with bowtie2 v2.2.9 (48). Postalignment filtering was completed using the AQUAS pipeline (github.com/kundajelab/chipseq_pipeline). MACS2 v2.1.1.20160309 was used for peak calling with shift size values calculated from the SPP peak caller, and high-quality peaks identified using the idr1 pipeline (idr score less than or equal to 0.02) (49, 50). Peak annotations, gene ontology analyses, motif identification, and binding heat maps were identified using HOMER v4.9 (51).

### qPCR

RNA was extracted using the RNeasy Mini Kit (QIAGEN). cDNA was prepared using iScript Reverse Transcription Supermix (Bio-Rad). qPCRs were run using SYBR Green qPCR Master Mix (Applied Biosystems) on a StepOnePlus Real-Time PCR machine (Applied Biosystems). All qPCRs were standardized using Actin. Primers for specific genes are listed in Table S2.

### miRNA qPCRs

miR-30a levels were analyzed using the TaqMan Reverse Transcription kit (Applied Biosystems) and TaqMan MicroRNA Assays (Applied Biosystems). qPCR analysis was run using TaqMan Universal Master Mix II (no UNG) (Applied Biosystems) on a StepOnePlus Real-Time PCR machine. All miR-30a levels were standardized to U6.

### RNA-Seq library preparation, sequencing, and analysis

Total RNA integrity was assessed using the Agilent RNA 6000 Nano kit (Cat No. 5067-1511) on the Bioanalyzer. Hundred nanograms of total RNA (RNA integrity number values above 9) was used to prepare the total RNA-Seq libraries using the Illumina TruSeq Stranded Total RNA with Ribo-Zero Human (Cat No. RS-122-2201). Libraries were quantified using Qubit 2.0 Fluorometer (Life Technologies), and the quality of the libraries was assessed with Agilent High Sensitivity DNA kit (Cat No. 5067-4626). Libraries were sequenced on the Illumina NextSeq 500 platform with NextSeq 500 High Output v2 sequencing kit (Cat No. FC-404-2002). Reads were aligned to the hg19 genome build using HiSAT2 v 2.1.0 with the --dta-cufflinks command, assembled into transcripts with StringTie (v1.3.3), cuffquant (v and cuffdiff (v 2.2.1)) to determine differentially expressed genes (52).

### CRISPR YAP −/− and miR-30a KO cell lines

For YAP −/−, guide RNA (TCCGGACCCGGGCAACCG) targeting the first exon of YAP was cloned into the PX459 plasmid. These cells have been previously characterized (8). For miR-30a KO, guide RNA (GCCACAGATGGGCTTTCAGT) targeting the loop region of miR-30a was cloned into PX459. hSC2λ cells were transfected with each plasmid and treated with 0.25 μg/ml puromycin (Gibco) for 48 h. Single clones were selected, expanded, and subjected to Western blot analysis (YAP −/−) or miRNA-qPCR analysis (miR-30a KO). Editing of the region of interest was assessed by Sanger sequencing for both cell lines.

### Luciferase assay

3′UTRs from *PTPN13* and *KLF9* were cloned into the pmirGLO Dual-Luciferase miRNA Target Expression Vector (Promega). miR-30a binding sites were mutated using the Q5 Site-Directed Mutagenesis Kit (NEB). Mutagenesis primers are listed in Table S1. hSC2λ cells were transfected and seeded in opaque 96-well plates, six replicates for each condition. Twenty-four hours after seeding, luciferase activity was measured with the Dual-Luciferase Assay System (Promega) according to manufacturer’s instructions. Firefly luciferase signal was normalized with the *Renilla* luciferase signal.

### Growth assay

hSC2λ cells were plated in 12-well plates, 10,000 cells per well, in triplicate (15 wells total). Cells were counted at the same time each day using a hemocytometer for 5 days. For the HEI-193 assays, cells were plated 20,000 cells per well in 12-well plates in triplicate and counted every other day for 10 days to account for the slower growth phenotype. Growth curves were created using GraphPad Prism (version 8).

### Cell cycle analysis

hSC2λ WT or miR-30a KO cells were plated in 10-cm dishes, at 500,000 cells per plate, in triplicate. Cells were collected 72 h later and fixed in 70% ethanol for 24 h. Cells were stained with propidium iodide (#P3566, Invitrogen) at 20 μg/ml. Propidium iodide was measured by flow cytometry. The cell cycle was determined using FlowJo (V10.7).

### Cleaved caspase-3 assay

hSC2λ and miR-30a KO cells were plated in 10-cm dishes, at 500,000 cells per plate, in triplicate. Cells were collected 72 h later for flow cytometry analysis. Cells were stained with Zombie Violet Fixable Viability dye (#423113, BioLegend) and with FITC conjugated Active Caspase 3 (#550480, BD PharMingen).

### Statistical analysis

Statistical analysis of data was performed using GraphPad Prism (version 8). Individual statistical methods are described in the respective figure legends and were conducted using the data included in the figures. All experiments were conducted three times (n = 3). Unpaired Student’s *t* test was used to determine the significance of the results and the two-tailed *p* values. Unless otherwise noted, the mean and SD were used to assess the significance. Significance was determined for growth assays with more than two treatment groups using full factorial three-way ANOVA. Data sets were normalized using y=sqrt(Y). Post hoc analysis was conducted where ANOVA was significant and is indicated in the figure legends. All *p*-values and degrees of freedom from ANOVA are reported in Table S7.

## Data availability

Some of the data used in this study are deposited into publicly available databases. The ChIP-seq data are stored in the Gene Expression Omnibus under accession number GSE112932. RNA-Seq data are stored in the Gene Expression Omnibus under accession number GSE163079.

## Supporting information

This article contains supporting information.

## Conflict of interest

The authors declare that they have no conflicts of interest with the contents of this article.
