# Supplementary material for: Transcriptional regulation of miR-30a by YAP impacts PTPN13 and KLF9 levels and Schwann cell proliferation
Source: J Biol Chem. 2021 Jul 12;297(2):100962. doi: 10.1016/j.jbc.2021.100962 (PMC8348554; doi:10.1016/j.jbc.2021.100962)
Supplement: Supplemental Figures S1–S7 [file mmc1.docx]

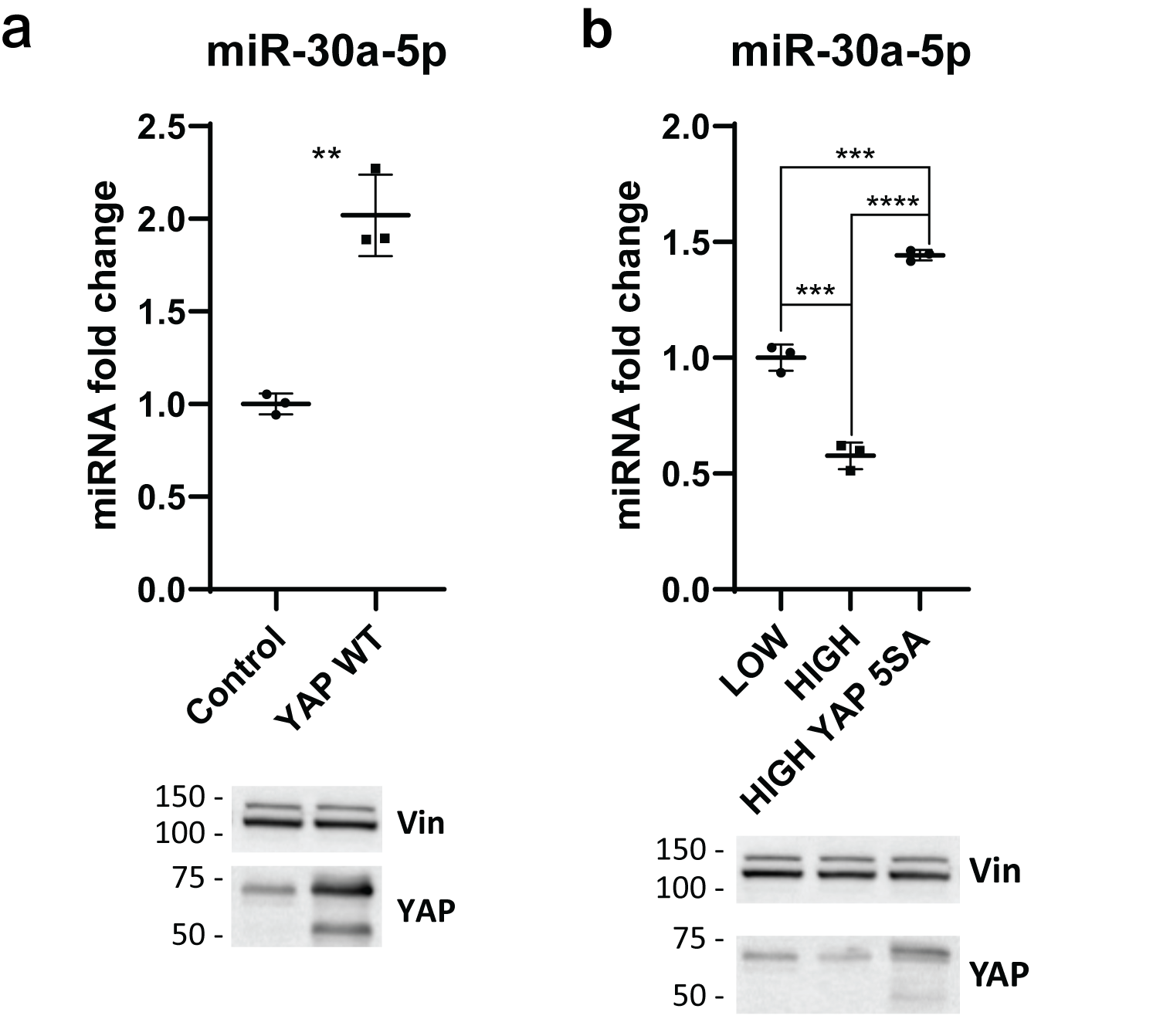


**Figure S1. Regulation of miR-30a through YAP.** (**a**) Analysis of miR-30a expression levels in hSC2λ cells with doxycycline transducible YAP WT overexpression. YAP WT indicates cells treated with 2μg/ml doxycycline to upregulate YAP expression. Replicates are indicated by separate data points. Western blot confirms upregulation of YAP. Vinculin (vin) used as a loading control. Molecular weight markers are indicated in kDa (n=3; ** = p<0.01, two-tailed Student’s t test; error bars = SD). (**b**) Rescue of miR-30a downregulation at high density by overexpression of YAP-5SA. Replicates are indicated by separate data points. Western blot confirms downregulation of YAP at high density and subsequent overexpression of YAP with YAP-5SA transfection. Vinculin (vin) used as a loading control. Molecular weight markers are indicated in kDa (n=3; *** = p<0.001, **** = p<0.0001, two-tailed Student’s t test; error bars = SD). (**a-b**) qPCRs are representative of three individual experiments with three replicates each.


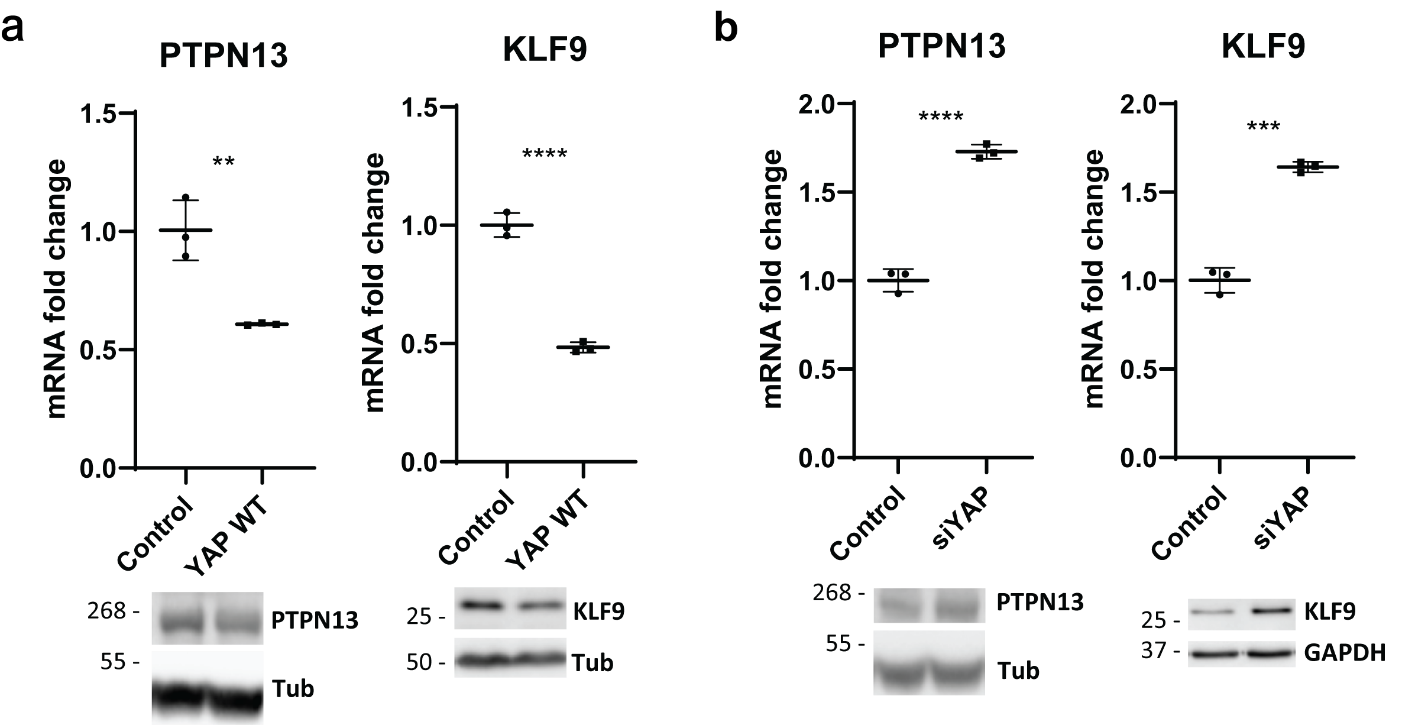


**Figure S2. Regulation of PTPN13 and KLF9 through YAP.** (**a**) Analysis of PTPN13 and KLF9 expression levels in hSC2λ cells with doxycycline transducible YAP WT overexpression. YAP WT indicates cells treated with 2μg/ml doxycycline to upregulate YAP expression. Replicates are indicated by separate data points. Upregulation of YAP is confirmed in Figure S1a. Downregulation of targets is confirmed by Western blot using Tubulin (Tub) as a loading control. Molecular weight markers are indicated in kDa (n=3; ** = p<0.01, ****=p<0.0001, two-tailed Student’s t test; error bars = SD). (**b**) Analysis of PTPN13 and KLF9 expression in hSC2λ cells transfected with siRNA non-targeting control or siYAP. Replicates are indicated by separate data points. Downregulation of YAP is confirmed in Figure 1f. Western blot analysis confirms upregulation of PTPN13 and KLF9 expression upon suppression of YAP. Tubulin (Tub) and GAPDH used as a loading controls. Molecular weight markers are indicated in kDa (n=3; *** = p<0.001, **** = p<0.0001, two-tailed Student’s t test; error bars = SD). (**a-b**) qPCRs are representative of three individual experiments with three replicates each.


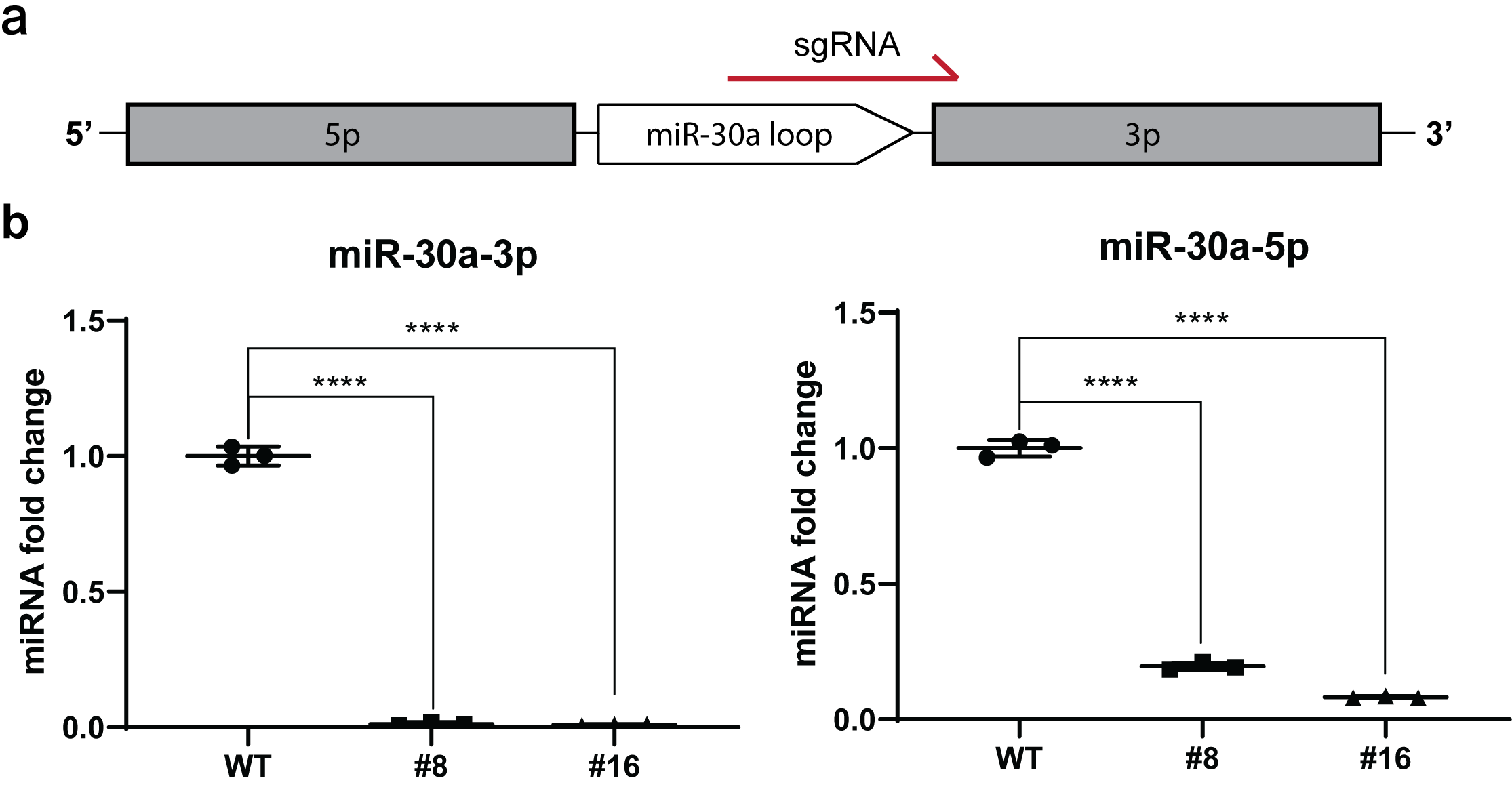


**Figure S3. Generation of miR-30a KO through CRISPR/Cas9.** (**a**) Schematic representation of the guide RNA placement for CRISPR/Cas9. (**b**) Confirmation by miRNA qPCR of loss of miR-30a. WT indicates hSC2λ control cells. #8 and #16 indicate two different hSC2λ clones with loss of miR-30a (n=3; **** = p<0.0001, two-tailed Student’s t test; error bars = SD).


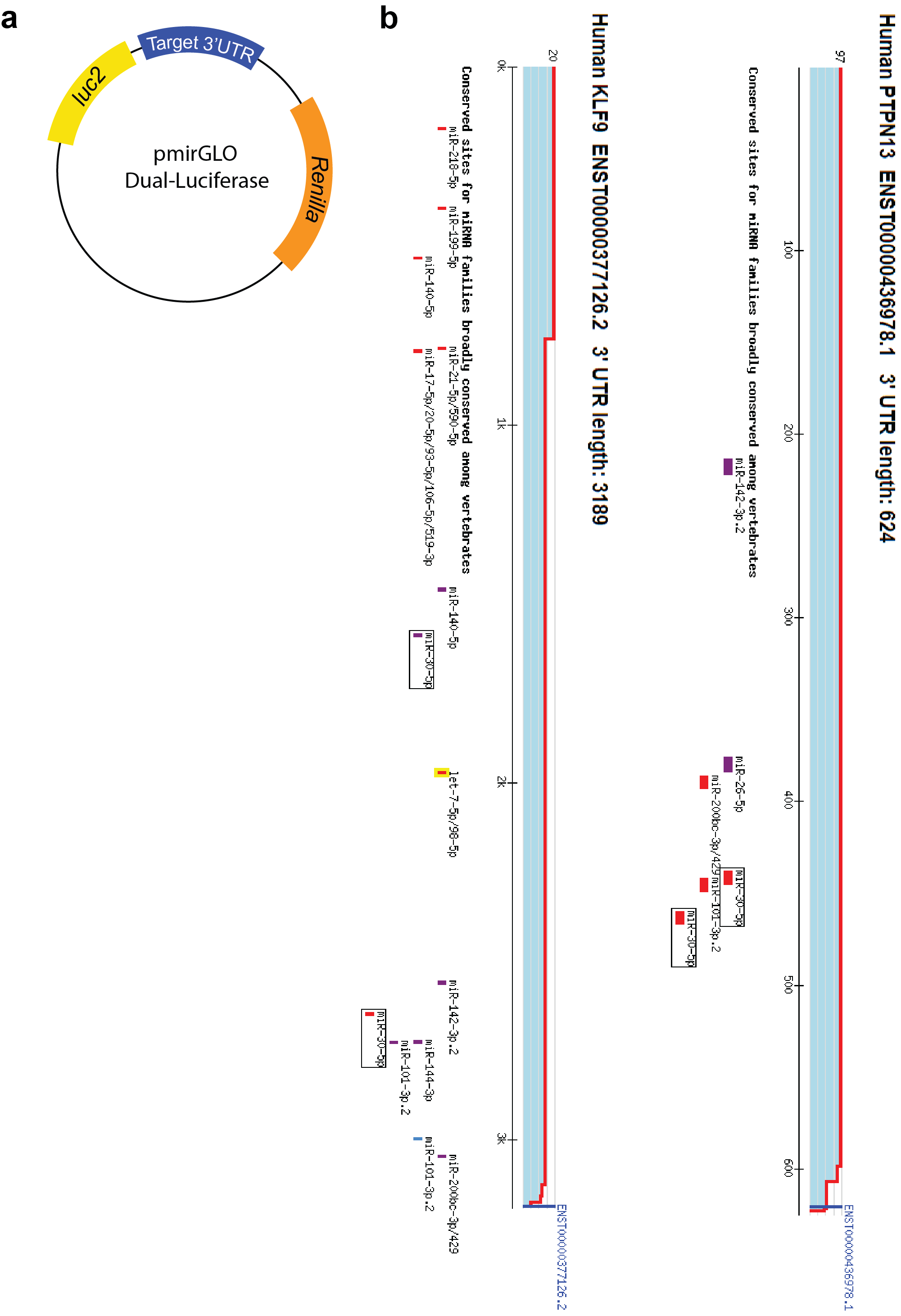


**Figure S4. Schematic representations pmirGLO vector and the 3’UTRs of PTPN13 and KLF9.** (**a**) Simplified schematic for the pmirGLO vector. 3’UTRs were cloned downstream of *luc2* luciferase gene. *Renilla* luciferase is used as an internal standard. (**b**) Representations of PTPN13 and KLF9 3’UTRs. miRNA binding sites are determined with TargetScan (targetscan.org). miR-30a-5p binding sites are indicated in red and highlighted in boxes.

**
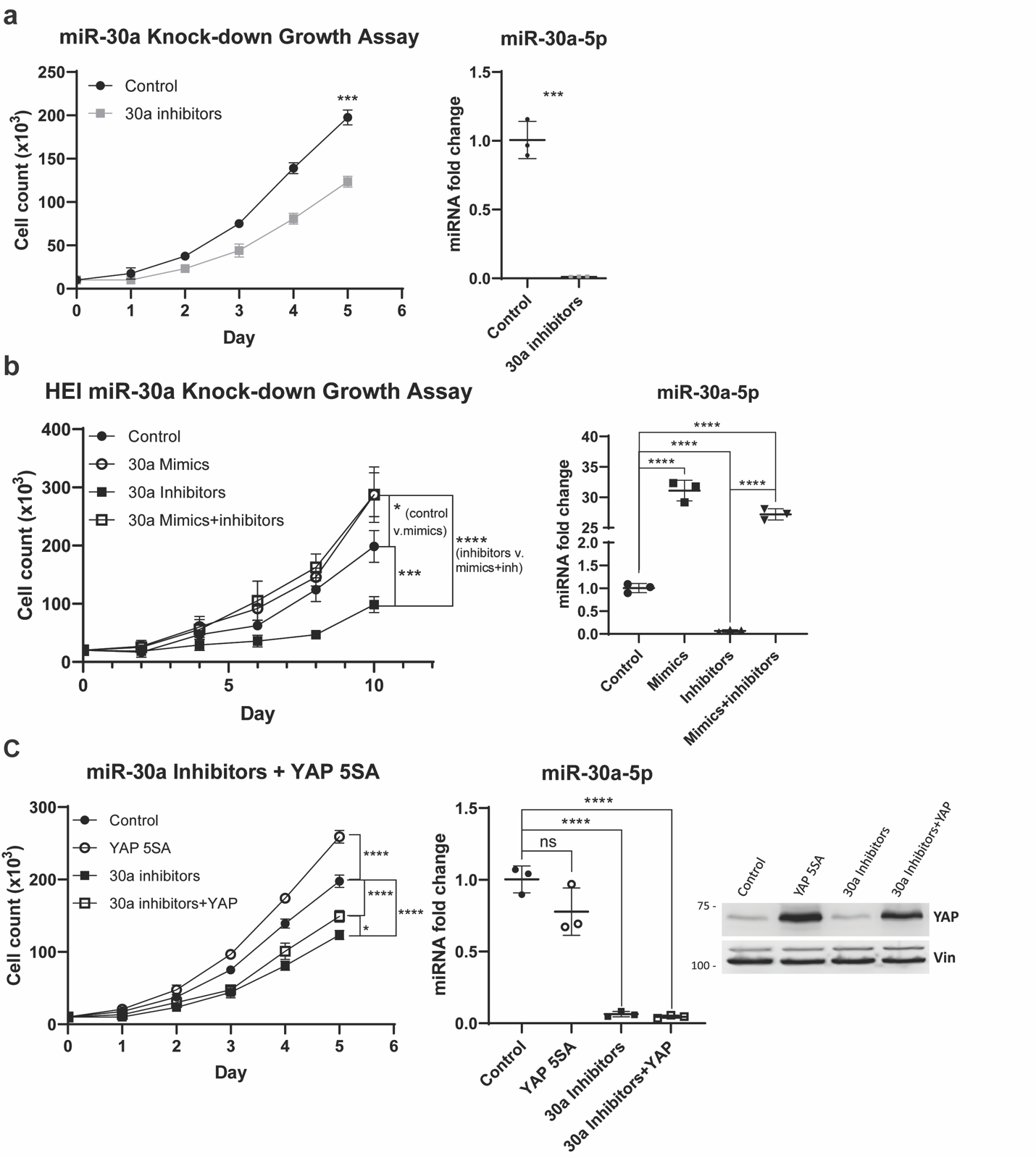
**

**Figure S5. Effects of miR-30a knock-down on cellular growth in hSC2**λ **and HEI-193 cells.** (**a**) miR-30a was knocked-down in hSC2λ cells using miRNA inhibitors (gray squares). Control indicates non-targeting control (black circles). Growth rates were assessed over five days by cell counting. Significance was calculated for day five. miRNA qPCR confirms significant knock-down of miR-30a in hSC2λ cells (n=3; *** = p<0.001, two-tailed Student’s t test; error bars = SD). (**b**) HEI-193 cells were transfected with non-targeting controls, miR-30a mimics, miR-30a inhibitors, or both miR-30a mimics + inhibitors. Growth rates were assessed over 10 days by cell counting. Significance was calculated using three-way ANOVA on transformed data. The differences in growth rates due to the interaction of genotype and treatment was significant (****, p-value < 0.0001). Post-hoc analysis was used to determine significance of day 10 values (n=3; * = p<0.05, *** = p<0.001, **** = p<0.0001; error bars = SD). miRNA qPCR analysis shows levels of miR-30a expression in the different treatment groups (n=3; **** = p<0.0001, two-tailed Student’s t test; error bars = SD). (**c**) hSC2λ cells were transfected with a control vector plus non-targeting miRNA control (Control), YAP-5SA overexpression vector and non-targeting miRNA control (YAP-5SA), control vector and miR-30a inhibitors (30a inhibitors), or YAP-5SA vector and miR-30a inhibitors (30a inhibitors+YAP). Growth rates were assessed over five days by cell counting. The differences in growth rates due to the interaction of genotype and treatment was significant (*, p-value = 0.0262). Post-hoc analysis was used to determine significance of day five values (n=3; * = p<0.05, **** = p<0.0001; error bars = SD). miRNA qPCR analysis shows levels of miR-30a expression in the different treatment groups (n=3; ns = not significant, **** = p<0.0001, two-tailed Student’s t test; error bars = SD). Western blot confirms overexpression of YAP. Vinculin used as a loading control. Molecular weight markers indicated in kDa. (**a-c**) Growth assays and qPCRs are representative of three individual experiments with three replicates each.

**
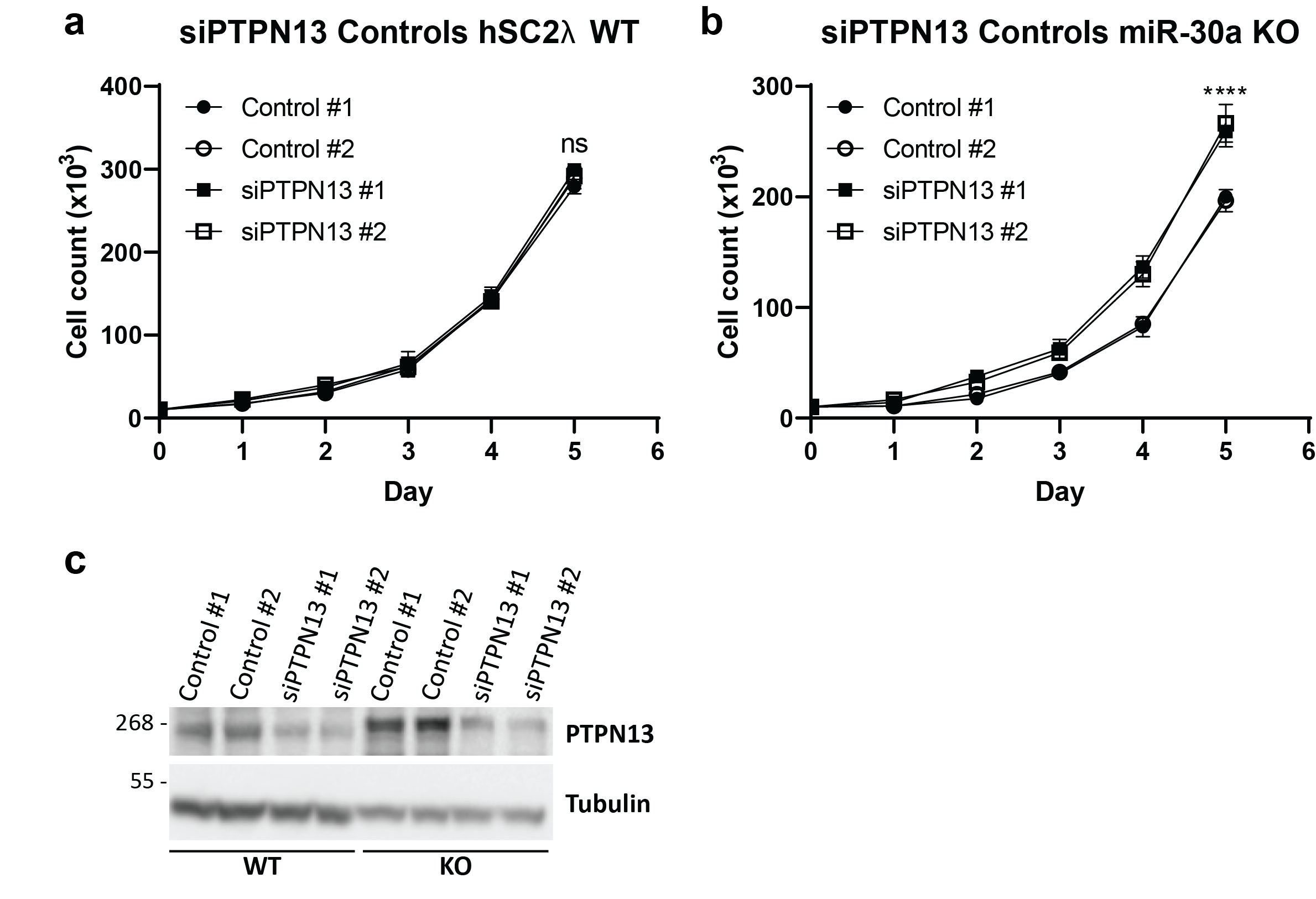
**

**Figure S6. Control growth assays for PTPN13 knock-down by siRNA.** (**a**) hSC2λ WT cells were transfected with individual non-targeting siRNA controls (Control #1 and #2) or individual siRNAs targeting PTPN13 (siPTPN13 #1 and #2). Growth rates were assessed over five days by cell counting. Significance was calculated using two-way ANOVA on transformed data. The effect of treatment on growth rates was not significant (p-value = 0.0859). (**b**) hSC2λ miR-30a KO cells were individual non-targeting siRNA controls (Control #1 and #2) or individual siRNAs targeting PTPN13 (siPTPN13 #1 and #2). Growth rates were assessed over five days by cell counting. Significance was calculated using two-way ANOVA on transformed data. The effect of treatment on growth rates was significant (****, p-value <0.0001). (**c**) Western blot confirms downregulation of PTPN13 using individual siRNAs. Tubulin used as a loading control. Molecular weight markers indicated in kDa. (**a-b**) Growth assays are representative of three individual experiments with three replicates each.


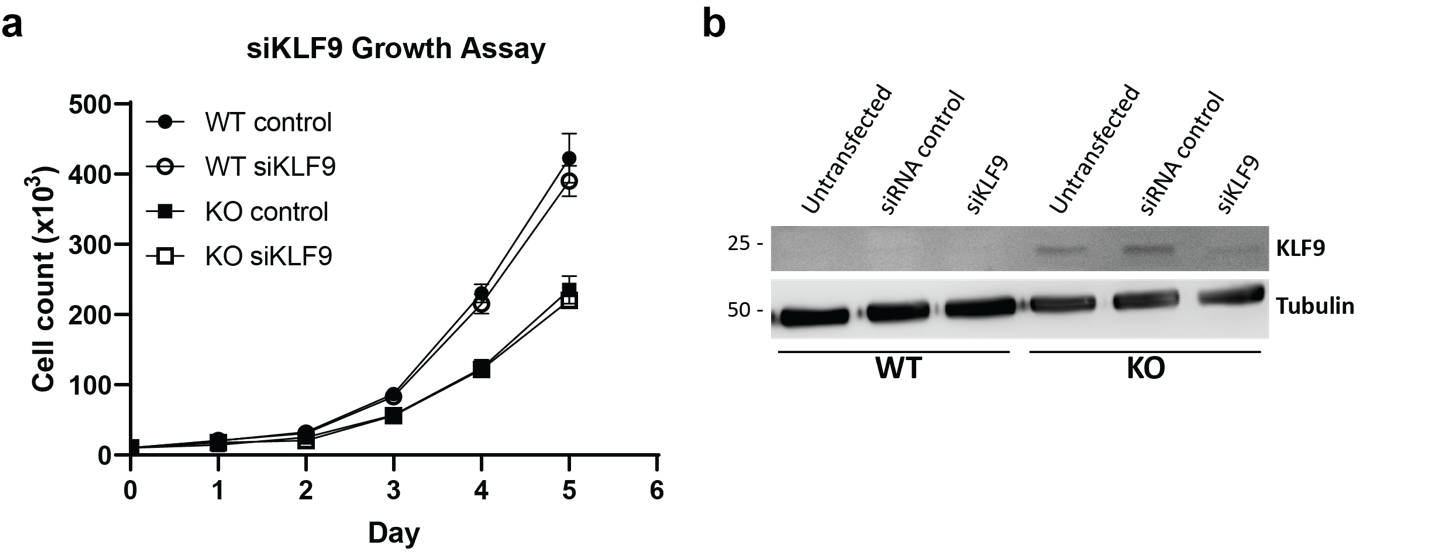


**Figure S7. Effect of knock-down of KLF9 on hSC2λ growth rates.** (**a**) hSC2λ WT and miR-30a KO cells were transfected with a non-targeting siRNA control (Control #1 and #2) or siRNA targeting KLF9. Growth rates were assessed over five days by cell counting. Significance was calculated using three-way ANOVA on transformed data. The effect of the interaction of genotype and treatment on growth rates was not significant (p-value = 0.458). Growth assay is representative of three individual experiments with three replicates each. (**b**) Western blot confirms downregulation of KLF9 using siRNA. Untransfected hSC2λ WT and miR-30a cells included as an additional control. Tubulin used as a loading control. Molecular weight markers indicated in kDa.
